# Circulating Activin A and Follistatin-like Proteins in Rheumatoid Arthritis with Interstitial Lung Disease: A Cross-Sectional Comparative Study

**DOI:** 10.3390/diagnostics16030399

**Published:** 2026-01-27

**Authors:** Firdevs Ulutaş, Kürşat Kaya, Nilüfer Yiğit, Veli Çobankara

**Affiliations:** 1 Division of Rheumatology, Department of Internal Medicine, Pamukkale University, 20160 Denizli, Turkey; 2 Division of Medical Biochemistry, Pamukkale University, 20160 Denizli, Turkey; 3 Division of Pulmonology, Pamukkale University, 20160 Denizli, Turkey

**Keywords:** rheumatoid arthritis, Activin A, Follistatin-like protein 1, Follistatin-like protein 3, interstitial lung disease, idiopathic pulmonary fibrosis

## Abstract

**Background/Objectives:** Rheumatoid arthritis-associated interstitial lung disease (RA-ILD) represents one of the major contributors to morbidity and mortality in Rheumatoid arthritis (RA), yet its underlying molecular mechanisms remain incompletely defined. Activin A, a member of the transforming growth factor-β (TGF-β) superfamily, has emerged as a key regulator of inflammation, fibroblast activation, and tissue remodeling. However, its role in RA patients with interstitial lung disease (ILD) has not been fully elucidated. We aimed to investigate circulating levels of Activin A, Follistatin-Like Protein-1 (FSTL1), and Follistatin-Like Protein-3 (FSTL3) in patients with RA, RA-ILD, idiopathic pulmonary fibrosis (IPF), and healthy controls and explore their associations with disease activity and pulmonary function parameters. **Methods:** This cross-sectional study included 90 participants: healthy controls (*n* = 20), RA (*n* = 25), RA-ILD (*n* = 21), and IPF (*n* = 24). Serum biomarkers were quantified using validated enzyme-linked immunosorbent assays (ELISAs). Clinical characteristics, inflammatory markers, disease activity indices, and pulmonary function tests were recorded. Group comparisons and correlation analyses were performed using appropriate parametric and non-parametric statistical methods. **Results:** Circulating Activin A levels were progressively increased from controls to RA, RA-ILD, and IPF, with significantly higher concentrations in all disease groups relative to controls. FSTL1 levels were significantly reduced in RA-ILD patients compared with RA and controls, while FSTL3 levels were markedly elevated in IPF. Activin A did not correlate with disease activity indices or pulmonary function parameters, whereas FSTL1 correlated positively with diffusing capacity of the lungs for carbon monoxide and disease duration, and FSTL3 showed an inverse association with lactate dehydrogenase. **Conclusions:** Activin A may be associated with the fibroinflammatory burden in both RA-ILD and IPF. The observation of altered circulating levels of Follistatin-like proteins—key regulatory molecules with multifaceted biological functions—suggests that the underlying pathogenesis is complex and governed by tightly regulated, interconnected signaling pathways.

## 1. Introduction

Rheumatoid arthritis (RA) is a chronic systemic inflammatory disease that most often develops in middle-aged women. While it primarily targets the synovium, the disease can also extend beyond the joints, with interstitial lung disease (ILD) being one of the most serious complications that increases both morbidity and mortality [1]. The mechanisms driving RA-ILD are not yet fully understood, but accumulating evidence suggests that fibrotic pathways are closely connected to the transforming growth factor-β (TGF-β) superfamily. Within this group, Activin A has gained attention as an important regulator of inflammation and tissue remodeling [2]. By modulating both T helper cell subsets and B-cell responses, it regulates inflammation, immunity, and fibrosis. In the context of fibrosis and tissue remodeling, it promotes fibroblast proliferation, extracellular matrix deposition, and scar formation [3]. In rheumatoid synovial tissue, fibroblast-like synoviocytes (FLSs) have been identified as primary producers of Activin A. Proinflammatory cytokines such as interleukin-1β (IL-1β), tumor necrosis factor-α (TNF-α), and TGF-β were shown to promote FLSs to synthesize Activin A [4]. These processes are tightly controlled by follistatin, the natural antagonist of Activin A. Follistatin-like proteins, such as Follistatin-like protein-3 (FSTL-3), function similarly to follistatin by antagonizing members of the TGF-β superfamily, mainly Activin A, and effectively blocks its profibrotic activity [5]. The regulatory axis between Activin A and FSTL-3 underscores the critical importance of fine-tuning this balance to restrain disease progression and mitigate tissue damage [6]. For a healthy immune response, and to protect the body from excessive systemic inflammation, maintaining this balance is essential [7].

Activin A is synthesized in the lung microenvironment, particularly by recruited alveolar macrophages (recAMs). Following lung injury, these macrophages engage a range of profibrotic signaling cascades that contribute to the development and progression of pulmonary fibrosis. Through its ability to facilitate epithelial–mesenchymal transition (EMT) and to amplify a self-sustaining profibrotic feedback mechanism within recAMs, Activin A plays a pivotal role in the fibrotic response after lung injury [8]. In idiopathic pulmonary fibrosis (IPF), patients who develop acute exacerbations have been reported to exhibit markedly higher circulating Activin A concentrations than those with stable disease. Increased Activin A levels were strongly linked to both the onset of exacerbations and subsequent clinical worsening [9]. Beyond pulmonary pathology, the role of Activin A has also been explored in fibroinflammatory conditions such as systemic sclerosis (SSc). Evidence indicates that activation of the Activin A-dependent signaling axis promotes excessive collagen synthesis, thereby contributing to tissue fibrosis [10]. Moreover, Activin A has been shown to induce upregulation of xylosyltransferase-I (XT-I) in dermal fibroblasts, a mechanism that facilitates extracellular matrix accumulation in patients with SSc [11].

In view of these observations, we postulated that Activin A might contribute to the pathogenesis of RA-ILD in the context of RA. Given that the biological role of Activin A in RA and RA-ILD has not yet been fully elucidated, we sought to examine circulating Activin A levels, with particular emphasis on those with concomitant ILD. Such an approach may provide novel insights into the underlying pathobiological mechanisms of RA-ILD and support the identification of potential biomarkers for disease stratification and therapeutic targeting.

## 2. Materials & Methods

### 2.1. Study Design and Patient Selection

This cross-sectional study included a total of 90 participants. Patients with RA were consecutively enrolled from the rheumatology outpatient clinic of a tertiary referral center over the study period spanning May to July 2025. No randomization procedures were implemented. All patients met the established classification criteria for RA according to the 2010 American College of Rheumatology/European League Against Rheumatism (ACR/EULAR) criteria [12], as well as the diagnostic criteria for IPF defined in the 2018 American Thoracic Society, European Respiratory Society, Japanese Respiratory Society, and Latin American Thoracic Society (ATS/ERS/JRS/ALAT) clinical practice guidelines [13].

Individuals with other systemic autoimmune rheumatic diseases, fibrosing interstitial lung disorders unrelated to RA or IPF, active malignancy, chronic infections, or additional systemic inflammatory conditions with the potential to influence biomarker levels were excluded. Healthy control subjects were recruited from individuals without regular medication use and with no known history of chronic inflammatory or pulmonary diseases.

### 2.2. Determination of Diagnostic Tests and Disease Activity

All patients with RA underwent a systematic clinical evaluation. Individuals exhibiting respiratory symptoms suggestive of ILD, including chronic non-productive cough or exertional dyspnea, were further assessed with high-resolution computed tomography (HRCT) and pulmonary function testing during their outpatient visit. HRCT images were independently evaluated by an experienced pulmonologist who was blinded to all clinical, laboratory, and biomarker data. Patients demonstrating radiological features consistent with ILD were subsequently classified as having RA-ILD.

In addition, pulmonary function testing was performed in patients with IPF as part of routine clinical evaluation at the time of study inclusion. Assessed parameters included forced vital capacity (FVC), forced expiratory volume in one second (FEV1), the FEV1/FVC ratio, and diffusing capacity of the lung for carbon monoxide (DLCO). All tests were conducted using standardized spirometric methods and single-breath DLCO techniques in accordance with international recommendations. Pulmonary function outcomes were reported as percentages of predicted values, adjusted for age, sex, height, and ethnicity.

Comprehensive demographic information, including age, sex, and smoking status, as well as disease duration, comorbid conditions, and concomitant medication use, was systematically collected. Treatment-related data were retrospectively retrieved from electronic medical records at the time of study inclusion, and medication exposure was documented contemporaneously with serum sampling. Ongoing glucocorticoid (GC) use was defined as the presence of active systemic GC therapy at the time of blood collection and was recorded as a dichotomous variable (yes/no). Information on GC dose and treatment duration was therefore not incorporated into the analyses. Other antirheumatic therapies, including both conventional synthetic and biologic disease-modifying antirheumatic drugs (such as rituximab, tocilizumab, methotrexate, leflunomide, and others), were recorded as baseline characteristics; however, they were not included in the biomarker correlation analyses due to substantial therapeutic heterogeneity and the relatively limited sample size.

Serum levels of Activin A, FSTL1, and FSTL3 were measured using commercially available sandwich enzyme-linked immunosorbent assay (ELISA) kits (SunLong Biotech, Hangzhou, China), in strict accordance with the manufacturers’ instructions. All samples were analyzed in duplicate, and optical density was measured at 450 nm using a microplate reader. Concentrations were calculated from standard curves generated for each analyte, and final values were adjusted for the manufacturer-specified dilution factors.

Disease activity in patients with RA was assessed using the Disease Activity Score in 28 joints based on erythrocyte sedimentation rate (DAS28-ESR). A DAS28-ESR value of ≤3.2 was defined as remission or low disease activity, whereas values > 3.2 were classified as moderate or high disease activity [14].

### 2.3. Ethics Approval

Local ethical approval was obtained from Pamukkale University on 6 May 2025, under decision number 09.

### 2.4. Statistical Analysis

The data was analyzed using IBM SPSS Statistics version 31.0. Normality for continued variables in groups were determined by the Shapiro–Wilk test. The variance homogeneity of the values showing normal distribution was tested by Levene’s test. The variables that showed normal distribution and equal variance, were analyzed by Independent-Samples T test and One-Way ANOVA. Mean and standard deviation were used to summarize normally distributed data. The variables that didn’t show normal distribution were analyzed by Kruskal–Wallis test and Mann–Whitney U-test and those data are summarized as median (minimum- maximum values). The qualitative variables were analyzed by Chi-Square test and those data were presented as count. Relationships between parametric variables were investigated using the Pearson correlation test, and relationships between nonparametric variables and ordinal variables were investigated using the Spearman correlation test.

Point-biserial correlation analysis was applied to examine the relationships between continuous circulating biomarker concentrations (Activin A, FSTL1, and FSTL3) and binary clinical variables, including sex (male/female), rheumatoid factor (RF) status (positive/negative), anti-cyclic citrullinated peptide (anti-CCP) antibody status (positive/negative), and GC use at the time of serum sampling (yes/no). This statistical approach is appropriate for evaluating associations in which one variable is dichotomous and the other is continuous, allowing both the magnitude and direction of the relationship to be quantified. All statistical tests were conducted using a two-sided significance threshold of 0.05.

## 3. Results

### Study Population and Baseline Characteristics

The study comprised four groups with a total of 90 patients: healthy controls (*n* = 20), RA (*n* = 25), RA-ILD (*n* = 21), and IPF (*n* = 24). The control group was significantly younger than all disease groups (*p* < 0.001), whereas the IPF cohort exhibited a marked male predominance compared with the other groups (*p* = 0.041). No significant sex differences were observed between the RA and RA-ILD cohorts (Table 1).

When disease activity was evaluated, 12 of 21 RA-ILD patients (57.1%) had DAS28-ESR values > 3.2, whereas this proportion was markedly lower among RA patients without ILD, affecting only 5 of 25 individuals (20.0%). Conversely, remission or low disease activity (DAS28-ESR ≤ 3.2) was observed in 9 RA-ILD patients (42.9%) and in 20 RA patients (80.0%). This difference in disease activity distribution between groups was statistically significant (*p* = 0.014). Similarly, ESR was elevated in RA-ILD patients (*p* = 0.030), whereas C-reactive protein levels did not differ significantly between groups. Other biochemical parameters, including glucose, urea, creatinine, and liver function tests, were comparable (Table 1). Among the 46 patients with RA, rituximab was the most frequently used therapy (*n* = 24, 52.2%), followed by methotrexate (*n* = 5, 10.9%), leflunomide (*n* = 4, 8.7%), and tocilizumab (*n* = 3, 6.5%), while 21.7% of patients (*n* = 10) received other disease-modifying treatments. Overall, 56.5% (*n* = 26) of all RA patients were receiving concomitant GC therapy at the time of assessment.

Table 2 summarizes circulating levels of Activin A, FSTL1, and FSTL3 across the control, RA, RA-ILD, and IPF groups. Activin A concentrations differed significantly among groups, with the lowest levels observed in controls and progressively higher levels in RA, RA-ILD, and IPF patients. RA patients also exhibited lower Activin A concentrations than those with IPF. In contrast, the difference between RA and RA-ILD did not reach statistical significance. FSTL1 levels showed a distinct pattern, being significantly reduced in the RA-ILD group compared with both controls and RA patients, while RA patients had higher FSTL1 levels than IPF group. In contrast, FSTL3 levels were comparable between controls and RA patients but were significantly elevated in the IPF group compared with all other groups. Regarding FSTL-3, IPF patients had significantly higher serum levels than controls (*p* = 0.024), RA patients (*p* = 0.005), and RA-ILD patients (*p* < 0.001).

In the combined RA patient cohort (*n* = 46), when correlations were examined, Activin A did not show any significant associations with clinical, laboratory, or pulmonary function parameters. In contrast, FSTL1 demonstrated significant positive correlations with DLCO and disease duration, whereas FSTL3 was inversely correlated with lactate dehydrogenase (LDH) (Table 3).

Point-Biserial correlation analysis (*n* = 46) showed that Activin A levels were not associated with gender, RF or anti-CCP positivity, or GC use. This analysis demonstrated a significant negative association between GC use and both FSTL1 (rpb = −0.394, *p* = 0.007) and FSTL-3 levels (rpb = −0.535, *p* < 0.001). Overall, GC exposure was selectively associated with lower circulating FSTL1 and FSTL3 levels, whereas Activin A levels were unaffected (Table 4).

## 4. Discussion

To the best of our knowledge, this study represents one of the few clinical investigations to jointly evaluate RA, RA-ILD and IPF through the Activin A–FSTL3 axis. We believe that our work provides a meaningful contribution to the existing literature. In our analysis, serum Activin A levels were significantly elevated in patients with RA, RA-ILD and IPF compared with healthy controls, with the markedly elevated concentrations observed in the IPF group. The IPF group was older and demonstrated a clear male predominance, reflecting the known epidemiology of IPF [15]. It is well established that aging influences immune regulation and fibrotic pathways. In addition, sex-related differences in immune regulation and fibrotic responses may affect biomarker profiles and should be taken into account when interpreting study findings [16]. However, in the present study, circulating Activin A, FSTL1, and FSTL3 levels did not show significant correlations with age and gender-adjusted analyses. These findings suggest that the alterations observed in the Activin–follistatin axis are more likely related to disease-specific inflammatory and fibrotic processes rather than chronological aging alone.

In the recent literature, several profibrotic pathways—such as TGF-β, platelet-derived growth factor (PDGF), and Wnt/β-catenin signaling—have been shown to drive fibroblast proliferation and progressive interstitial fibrosis, orchestrated by pro-inflammatory cytokines. Importantly, these mechanisms are not limited to IPF but are also evident in connective tissue disease-associated ILD [17]. It appears evident that Activin A plays a pivotal role in this process, functioning as a member of the TGF-β superfamily. Elevated concentrations of Activin A in both serum and synovial fluid have previously been demonstrated in several inflammatory diseases, including RA. These increases were shown to correlate with markers of inflammation as well as overall disease activity [18]. In our cohort, patients with RA-ILD exhibited higher systemic inflammatory activity, reflected by increased DAS28-ESR scores and elevated ESR compared with patients with RA without ILD. Despite this difference, Activin A concentrations were comparable between the two groups. The stepwise increase in circulating Activin A levels from healthy controls to RA, RA-ILD, and IPF suggests a relationship with an escalating inflammatory–fibrotic disease spectrum. However, the lack of significant associations with disease activity indices, inflammatory markers, or pulmonary function parameters indicates that Activin A is unlikely to reflect contemporaneous clinical activity. Instead, Activin A may represent a marker of cumulative or global inflammatory–fibrotic burden rather than short-term disease fluctuations. This distinction is particularly relevant in cross-sectional analyses and underscores the need for longitudinal studies to determine whether changes in Activin A levels track disease progression or therapeutic response over time.

Although members of the Follistatin-like protein family share conserved structural domains, each isoform fulfills a unique biological role. Together, these proteins operate within a finely balanced regulatory network that orchestrates the interplay between inflammatory signaling, physiological tissue repair, and the transition toward pathological fibrotic remodeling [19]. FSTL1 exerts a broad range of biological effects, positioning it as a key modulator of tissue homeostasis [20]. FSTL1 is upregulated following lung injury and contributes to myofibroblast accumulation and fibrotic remodeling, indicating its potential as a therapeutic target in progressive pulmonary fibrosis [21]. In patients with silicosis, FSTL1 also promotes fibrotic progression through the positive regulation of TGF-β1 signaling pathways [22]. In contrast, FSTL3 appears to function primarily as a regulatory component within the Activin A–SMAD signaling axis, modulating excessive profibrotic signaling rather than reflecting inflammatory burden per se [6]. In our cohort, the observation of reduced FSTL1 levels in patients with RA-ILD alongside markedly elevated FSTL3 levels in IPF may appear counterintuitive at first glance. However, this apparent divergence is more plausibly explained by stage-specific modulation of fibrotic signaling rather than by contradictory biological functions that may reflect differential regulation of the Activin–follistatin axis across disease stages. FSTL1 has been implicated in inflammation-associated tissue responses and early fibro-inflammatory processes, whereas FSTL3 may be preferentially upregulated in advanced, inflammation-independent fibrotic remodeling. Although this stage-dependent interpretation is biologically plausible, it is not directly supported by longitudinal follow-up or imaging-based fibrosis staging in the present study and should therefore be considered hypothesis-generating. Supporting this stage-dependent interpretation, studies in patients with Familial Mediterranean Fever (FMF) have demonstrated alterations in FSTL1 levels, whereas FSTL3 concentrations remained comparable to those of healthy controls, suggesting that FSTL3 is not prominently engaged during predominantly inflammatory disease states [23]. Collectively, these findings suggest that FSTL1 and FSTL3 mark distinct temporal and mechanistic stages of pulmonary fibrosis, rather than representing conflicting biological signals. In our study, the concomitant observation of the highest circulating Activin A levels together with markedly elevated FSTL3 concentrations in patients with IPF may reflect a compensatory counter-regulatory response aimed at attenuating Activin A-driven profibrotic signaling.

In the absence of treatment stratification or adjustment, the observed differences in circulating Activin A, FSTL1 and FSTL3 cannot be attributed solely to underlying disease biology. Pharmacological immunomodulation—particularly in patients with RA-ILD, in whom therapeutic strategies frequently differ—may have independently shaped immune–fibrotic biomarker profiles. Accordingly, these findings should be interpreted cautiously and regarded as hypothesis-generating rather than definitive. Nevertheless, a notable observation of the present study is that GC use was consistently associated with lower circulating levels of FSTL1 and FSTL3, whereas no such relationship was identified for Activin A. This pattern is biologically plausible, given that FSTL1 expression is tightly regulated by pro-inflammatory cytokines and tissue injury-related signals, both of which are effectively attenuated by GC therapy [24]. Consistent with this mechanism, FSTL1 has been shown to be declined following anti-inflammatory or immunosuppressive treatment, supporting its role as an inflammation-responsive biomarker [19]. LDH has been recognized as a prognostic biomarker in a range of pulmonary disorders, including lung malignancies, and its clinical relevance is well established in progressive pulmonary fibrosis, where elevated levels are associated with adverse outcomes [25]. Our findings indicate that FSTL3 levels were most pronounced in patients with IPF and were inversely associated with circulating LDH concentrations.

## 5. Limitations and Strengths

This study has several limitations. Given the cross-sectional design, limited sample size, and treatment heterogeneity, causal interpretation of biomarker differences is not possible, and the present findings should be considered exploratory and hypothesis-generating. An important limitation of this study is the lack of detailed treatment-related data, cumulative GC dose, and treatment duration. Therefore, the potential influence of specific therapies and cumulative treatment exposure on circulating biomarker levels cannot be excluded and should be addressed in future longitudinal studies.

The relatively small sample sizes, particularly in the RA and RA-ILD subgroups, may have limited statistical power and increased the risk of type II error. Consequently, the lack of statistically significant differences or correlations—especially for Activin A—should be interpreted cautiously and does not exclude the possibility of modest but biologically relevant associations.

Another important methodological limitation is the potential interference of RF in sandwich ELISAs, as RF may bind to the Fc region of assay antibodies and generate falsely elevated signals. In this study, no RF-blocking procedures or heterophilic antibody controls were applied, and assay performance was not specifically validated in RF-positive samples. Therefore, analytical interference related to RF cannot be fully excluded. Although no significant associations were observed between RF positivity and circulating biomarker levels, this finding does not preclude subtle assay interference.

Despite these constraints, the study has notable strengths, including the use of well-defined comparator groups, the concurrent evaluation of Activin A and its endogenous regulators (such as FSTL3), and the application of standardized disease activity measures and pulmonary function testing. By integrating rheumatologic and pulmonary perspectives, this work addresses a relevant knowledge gap in RA-ILD biomarker research.

## 6. Conclusions

Alterations in the Activin A–follistatin regulatory axis appear to be a defining feature of RA-ILD and IPF. The observed elevation of Activin A in both RA-ILD and IPF may be related to systemic inflammatory and fibrotic burden. Concurrently, the differential expression patterns of Follistatin-like proteins in these conditions suggest that they may actively participate in disease pathogenesis. However, the divergent and sometimes opposing biological functions of these proteins indicate that their effects are mediated through complex, tightly regulated signaling networks rather than linear pathways.

## Figures and Tables

**Table 1:** Demographic, laboratory, disease activity, and pulmonary function characteristics of the whole study groups.

Variable	Control (*n* = 20)	RA (*n* = 25)	RA-ILD (*n* = 21)	IPF (*n* = 24)	*p* Value
Age (years)	34.55 ± 11.22 *	62.76 ± 9.12	65.61 ± 9.92	68.29 ± 7.77	<0.001
Gender, male/female (*n*)	14-Jun	17-Aug	15-Jun	17 **/7 ***	<0.001
Disease Duration (year)	–	7 (1–30)	8 (1–32)	–	0.921
Glucose (mg/dL)	–	94 (73–159)	100 (78–205)	–	0.612
Urea	–	25 (20–62)	32 (22–93)	–	0.063
Creatinine	–	0.74 (0.40–1.60)	0.90 (0.50–2)	–	0.464
White blood cell count (/mm^3^)	–	8.316 ± 2142	8.382 ± 2.931	–	0.931
Platelet count (/mm^3^)	–	275.360 ± 68.839	285.380 ± 83.493	–	0.658
Hemoglobin (g/dL)	–	13.29 ± 1.33	12.55 ± 2.25	–	0.177
AST (U/L)	–	18.32 ± 5.87	17.85 ± 6.68	–	0.804
ALT (U/L)	–	16.00 (8.00–38.00)	15.00 (7.00–27.00)	–	0.208
LDH (U/L)	–	199.68 ± 47.50	230.04 ± 69.40	–	0.086
C-reactive protein (mg/L)	–	1.80 (0.30–12.00)	4.30 (0.30–32.00)	–	0.115
Erythrocyte sedimentation rate (mm/h)	–	16.00 (3.00–48.00)	25.00 (3.00–89.00) *	–	0.03
Remission or low disease activity (DAS28-ESR ≤ 3.2) (*n*, %)	–	20 (80%)	9 (42.9%)	–	0.014
Moderate to high disease activity (DAS28-ESR > 3.2) (*n*, %)	–	5 (20%)	12 (57.1%)	–	
FEV1 (%)	–	91.33 ± 12.20	79.28 ± 21.69 *	–	0.031
FVC (%)	–	93.32 ± 16.93	80.52 ± 22.14 *	–	0.032
DLCO (%)	–	83.84 ± 14.60	60.42 ± 12.60 *	–	<0.001

Abbreviations: RA, rheumatoid arthritis; RA-ILD, rheumatoid arthritis–interstitial lung disease, IPF, idiopathic pulmonary fibrosis; FEV1, forced expiratory volume in 1 s; FVC, forced vital capacity; DLCO, diffusing capacity of carbon monoxide; DAS28-ESR, Disease Activity Score in 28 joints using erythrocyte sedimentation rate; AST, aspartate aminotransferase; ALT, alanine aminotransferase; LDH, lactate dehydrogenase. Asterisks (*) denote values significantly different from the RA group, whereas double (**) and triple (***) asterisks indicate statistically significant male and female predominance, respectively, in the IPF group.

**Table 2:** Circulating Levels of Activin A, FSTL1, and FSTL3 across study groups.

Biomarker	Control (*n* = 20)	RA (*n* = 25)	RA-ILD (*n* = 21)	IPF (*n* = 24)	Significant Between-Group Differences
Activin A (pg/mL)	285 ± 38	321 ± 62	339 ± 55	358 ± 65	Control < RA, RA-ILD, IPF; RA < IPF
FSTL1 (ng/mL)	21.70 ± 6.22	23.02 ± 8.21	16.96 ± 8.01	18.19 ± 7.09	RA-ILD < Control, RA; RA > IPF
FSTL3 (pg/mL)	388 ± 111	388 ± 93	352 ± 49	485 ± 95	IPF > Control, RA, RA-ILD

Variables are summarized as mean ± standard deviation. Abbreviations: RA, rheumatoid arthritis; RA-ILD, rheumatoid arthritis with interstitial lung disease; IPF, idiopathic pulmonary fibrosis; FSTL1, Follistatin-Like Protein-1; FSTL3, Follistatin-Like Protein-3. Group comparisons were performed using one-way ANOVA with post hoc multiple-comparison testing. Only statistically significant pairwise differences (*p* < 0.05) are shown.

**Table 3:** Significant correlations of Activin A, FSTL1, and FSTL3 (*n* = 46) in all RA patients.

Biomarker	Variable	Correlation Coefficient (r)	*p* Value
FSTL1	DLCO (%)	0.35	0.017
FSTL1	Disease duration (years)	0.31	0.039
FSTL3	LDH (U/L)	−0.30	0.041

Correlation coefficients were calculated using Spearman’s rank correlation analysis. Only statistically significant correlations (*p* < 0.05) are shown. Abbreviations: Follistatin-Like Protein-1 (FSTL1), Follistatin-Like Protein-3 (FSTL3), DLCO, diffusing capacity of carbon monoxide; LDH, lactate dehydrogenase.

**Table 4:** Point-Biserial correlations between biomarkers and clinical variables in all RA patients (*n* = 46).

Biomarker	Statistic	Gender	RF Positivity	Anti-CCP Positivity	GC Use
Activin A	r_pb_	0.176	0.114	−0.040	−0.004
	*p*	0.242	0.449	0.790	0.981
FSTL1	r_pb_	−0.055	−0.237	−0.015	−0.394
	*p*	0.718	0.112	0.920	0.007
FSTL3	r_pb_	0.021	−0.078	−0.053	−0.535
	*p*	0.889	0.604	0.726	<0.001

Abbreviations: FSTL1, Follistatin-Like Protein-1; FSTL3, Follistatin-Like Protein-3; rpb point-biserial correlation coefficient; RF, rheumatoid factor; anti-CCP, anti-cyclic citrullinated peptide antibody; GC, glucocorticoid.

## Data Availability

The original contributions presented in this study are included in the article. Further inquiries can be directed to the corresponding author.
